# Identification of the Campanian Ignimbrite in the Dead Sea and consequent time-transgressive hydroclimatic shifts in the Eastern Mediterranean

**DOI:** 10.1038/s41598-024-59639-7

**Published:** 2024-05-27

**Authors:** Rebecca J. Kearney, Markus J. Schwab, Daniel Redant, Ina Neugebauer, Oona Appelt, Cecile Blanchet, Jan Fietzke, Christina Günter, Daniela J. M. Müller, Rik Tjallingii, Achim Brauer

**Affiliations:** 1 Section ‘Climate Dynamics and Landscape Evolution’, GFZ German Research Centre for Geosciences, Potsdam, Germany; 2 Section ‘Chemistry and Physics of Earth Materials’, GFZ German Research Centre for Geosciences, Potsdam, Germany; 3 Section ‘Geomorphology’, GFZ German Research Centre for Geosciences, Potsdam, Germany; 4 GEOMAR Helmholtz Centre for Ocean Research Kiel, Kiel, Germany; 5 Institute of Geosciences, University of Potsdam, Potsdam, Germany; 6 Institute of Earth Sciences, Heidelberg University, Heidelberg, Germany

**Keywords:** Palaeoclimate, Environmental sciences

## Abstract

Robust chronologies and time equivalent tephra markers are essential to better understand spatial palaeoenvironmental response to past abrupt climatic changes. Identification of well-dated and widely dispersed volcanic ash by tephra and cryptotephra (microscopic volcanic ash) provides time synchronous tie-points and strongly reduces chronological uncertainties. Here, we present the major, minor and trace element analyses of cryptotephra shards in the Dead Sea Deep Drilling sedimentary record (DSDDP 5017-1A) matching the Campanian Ignimbrite (CI). This geochemical identification expands the known dispersal range of the CI to the southeastern Mediterranean, over 2300 km from the volcanic source. Due to the CI eruption occurring near-synchronous with North Atlantic ice surge of Heinrich Event 4 (HE4), this tephra provides insights into regional responses to large-scale climatic change in the Mediterranean. In the Dead Sea, the CI layer is associated with wetter climatic conditions. This contrasts with the contemporaneous occurrence of the CI deposition and dry conditions in the central and eastern Mediterranean suggesting a possible climate time-transgressive expansion of HE4. Our finding underscores the temporal and spatial complexity of regional climate responses and emphasises the importance of tephra as a time marker for studying large-scale climatic changes verses regional variations.

## Introduction

During the last glacial period, the Mediterranean region experienced several abrupt climatic shifts at centennial and millennial timescales^1,2^. Situated at the confluence of two contrasting climate systems^3,4^, this region is highly sensitive to climatic variability, particularly to changes in precipitation and evaporation. Dated palaeoclimatic archives from the region can provide valuable insight into the temporal and spatial environmental response to these past abrupt climatic shifts^5–7^. However, large age uncertainties and tuning to other records often prevent precisely pin-pointing leads, lags and feedback processes in the climatic system as well as determining the response of regional environments to climate shifts^8^.

Tephra shards that mark volcanic ash layers serve as a powerful chronological tool, enabling the refinement of tephrochronological age uncertainties and provide reliable isochronous tephrostratigraphic tie-points. Widely dispersed tephra from highly explosive eruptions help to facilitate precise temporal comparisons between palaeoenvironmental records over regional to continental scales^9,10^. Tephra layers are a particularly useful chronological tool when: (1) glass shards can be confidently correlated to a volcanic centre and/or eruption through chemical compositions^11^; (2) they are widely dispersed and provide tie-points in important records^12^; (3) well dated by absolute and relative dating techniques^13,14^ and; (4) they can be linked to a climatic/archaeological event or transition around the time of the eruption^8,15^.

**Figure 1:**
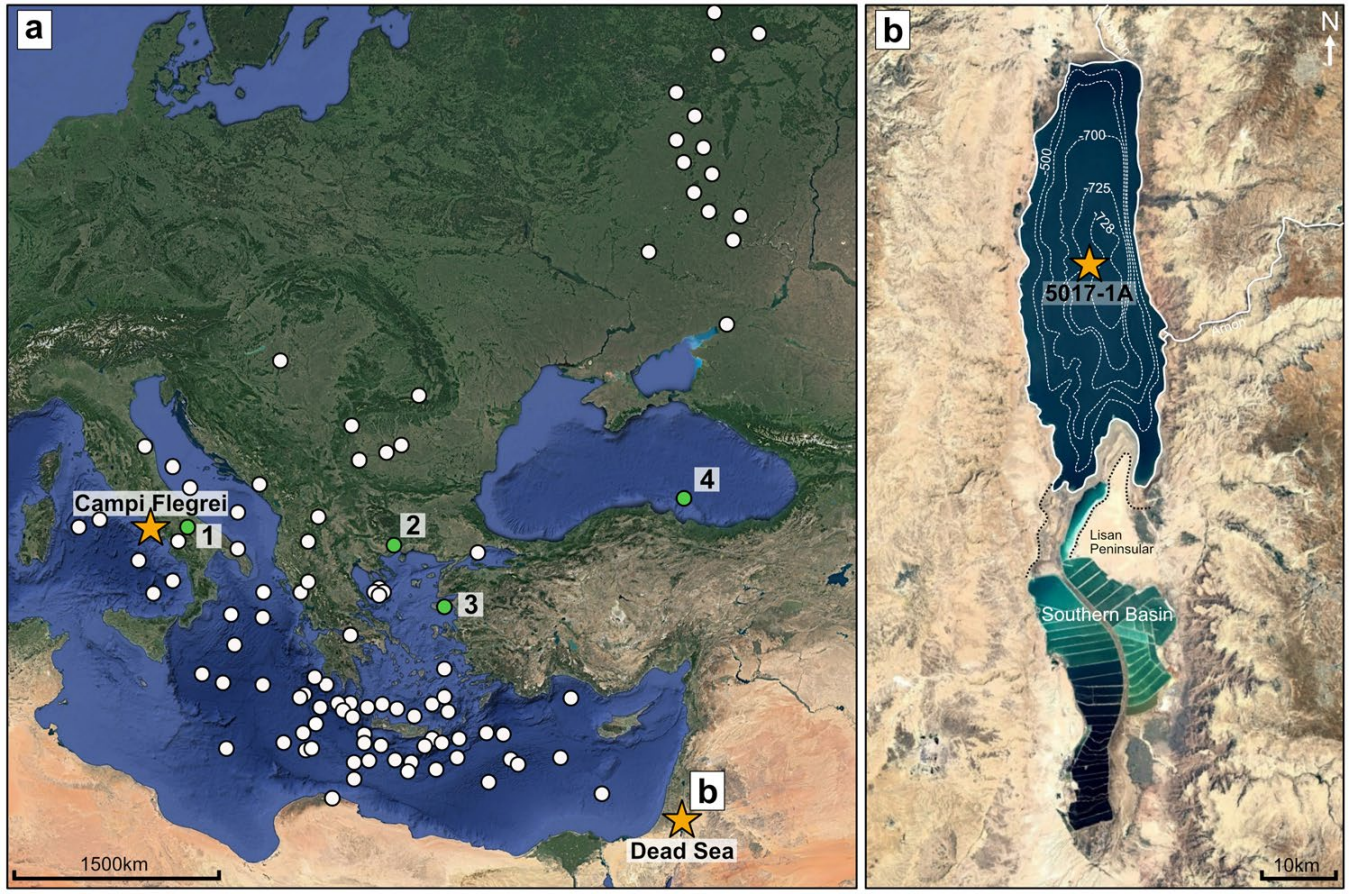
The location of the Dead Sea and sites that contain the Campanian Ignimbrite tephra within their records. (**a**) Map of the location of the Mediterranean region and eastern Europe showing the locations of records that have the Campanian Ignimbrite tephra within their sediments (updated from Smith et al.^18^). The sites highlighted in green are referred to in the text: (1) Lago Grande di Monticchio; (2) Tenaghi Philippon; (3) Megali Limni; (4) Black Sea M72-5-24-GC3. (**b**) A bathymetric map of the Dead Sea showing the location of the coring site, ICDP Dead Sea Deep Drilling Project 5017-1A (adapted from Müller et al.^5^). Satellite images used in both maps were obtained using Google Earth Pro application (version 7.3.6.9750, release date 2024) with data provided by SIO, NOAA, U.S. Navy, NGA, GEBCO and images from Landsat/Copernicus.

The Campanian Ignimbrite (CI), or the Y5 in marine records^16^, is seen as an important tephra layer. This caldera-forming eruption from Campi Flegrei, Italy is dated to 39.85 ± 0.14 ka^13^ (^40^Ar/^39^Ar, 2σ). The ash from this eruption was dispersed over 2000–3000 km across eastern Europe and the northern-central Mediterranean region^17^ (Fig. 1), deposited as visible and cryptotephra (non-visible) layers in numerous sedimentary archives in proximal^e.g.^^18–20^, distal marine^e.g.^^21–23^, terrestrial^e.g.^^24,25^ and archaeological sequences^e.g.^^17,26,27^ and has been extensively geochemically characterised. The CI is stratigraphically identified in many Mediterranean palaeoclimatic records to have occurred 700–800 years after a climatic downturn to colder and drier conditions in the Mediterranean region^21,28–30^, coinciding with the North Atlantic ice surge of Heinrich Event 4 (HE4)^31^. Even within the Greenland ice core records, a large SO_4_ spike in (North Greenland Ice Core Project (NorthGRIP) record with Greenland Stadial 9 is associated to the CI eruption^32^. In the archaeological record, the CI eruption deposits are close to the transition between Middle to Upper Palaeolithic cultures^26,33^.

The hypersaline Dead Sea, Levant (Fig. 1), provides a unique sedimentary record to identify the CI marker. Located at a pivotal location for climatic systems and the dispersal of past human populations between Africa and Eurasia^30^, this record can provide great insights into the role of climate on the local landscape and human history. Drilled as part of the International Continental Drilling Program (ICDP), the Dead Sea Deep Drilling (DSDDP) sedimentary record 5017-1A (Fig. 1), has undergone extensive palaeoenvironmental investigations^5,34,35^. Yet, large age uncertainties have hampered us to obtaining a detailed understanding of the temporal and spatial variability of past hydroclimate in the region^6,7,30^. Finding the CI layer in this important palaeoenvironmental record within a key archaeological region provides, not only an invaluable chronological marker to refine ages, but also enables direct correlations with other palaeoenvironmental and archaeological records throughout the Mediterranean region. Recent investigations have shown the preservation of cryptotephra within the sediments^6,36^ and with the current age model^7,37^, a targeted analysis can be undertaken to find the CI.

As a result, we present the discovery of the CI as a cryptotephra layer in the ICDP DSDDP 5017-1A record. This is the first time that this important tephra has been identified this far southeast in the Eastern Mediterranean region, providing an essential chronostratigraphic tie-point to verify the current chronology of the Dead Sea record, and allow direct linkages to other palaeoclimatic records in central and eastern Europe.

## Results

### Stratigraphy and lithology

**Figure 2:**
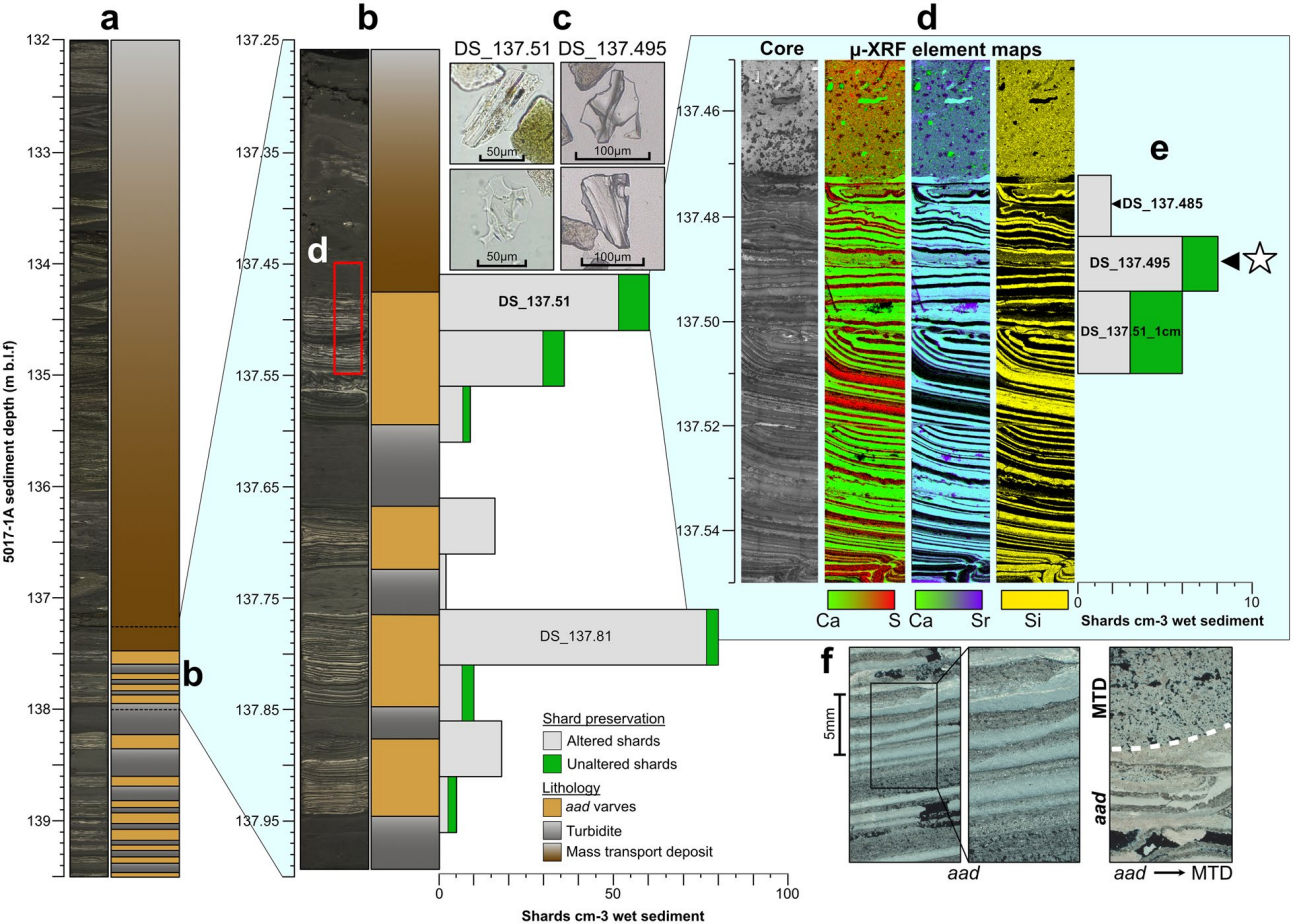
The stratigraphic context of the CI deposit in the ICDP core 5017-1A. (**a**) Overview of the DSDDP lithology that includes the *aad* section containing the CI and the thick MTD directly above. (**b**) Detailed view of the lithology and glass shard profile of the CI location. Low resolution sampling (5 cm) is shown. (**c**) Selected photos of glass shards identified in the sampled intervals. (**d**) Sample image and XRF element maps of a thin section at the depth of 137.45–137.55m blf (red box in **b**), which marks the *aad*-MTD transition. (**e**) High resolution sampling (1 cm) is shown. The tephra horizon analysed for glass shard geochemistry are highlighted and presented in this study as a white star. (**f**) Microscope images in polarized light of selected *aad* varves as well as the base of the MTD, showing an erosive contact to the underlying varved unit incorporating the CI and the overlying MTD.

The targeted sampling area encompassed a mass transport deposit (MTD) and undisturbed, finely laminated sediments interpreted as aragonite varves^38,39^ (Fig. 2). This MTD unit (thickness of ~ 5 m) is described as mud with few laminated layers that are highly folded (Fig. 2a), suggesting a major slumping event^39^.

Detailed examination of the transition from the laminated sediment to the MTD was examined using microscopic and geochemical analyses of a 10-cm long thin section (red box in Fig. 2a; at a depth of 137.45–137.55 m blf). The base of the massive unit in the upper 2 cm (~ 137.45–137.475 m blf) shows an undulating contact and clear traces of erosion of the underlying layers (Fig. 2f). In the Dead Sea basin, such non-laminated units are referred to as MTD in a broad sedimentological sense^39^. The laminated unit shows alternations of mm-thick aragonite-rich and detrital sub-layers typical of the *alternating aragonite detrital* (*aad*) facies in Dead Sea sediments (Fig. 2d), interpreted as annual laminations (varves)^40^. Two unconformities are identified at ca. 2.5–3 cm (~137.475 to 137.480 m blf) and at 5–5.5 cm (137.50 to 137.51 m blf, Fig. 2d) that might be related to erosive events or to the drilling of the core. The *aad* varves show some bending towards the top, just below the unconformities but are otherwise clearly defined and undisturbed, which suggests a quiet depositional environment. The layers between 6.7 and 7.4 cm (137.517–137.524 m blf) seem to have been disturbed by the emplacement of the MTD above them. The erosive contact identified between the *aad* varves and the overlying MTD precludes the use of the varved sequence to obtain additional chronological constrains as varves/annual layers may have been lost, and the extend of the loss cannot be estimated.

### Glass shard identification and geochemistry

Low resolution sampling (5 cm) was undertaken throughout the core sections to find volcanic glass shards as part of a pilot study (see “Methods”). Where a peak in glass concentration was found, higher sampling resolution (1–1.5 cm) was then undertaken to refine the depth of the true isochron. Samples are named according to the site name (Dead Sea, DS) and their lowest composite depth in metres (e.g., DS_mblf; see Supplementary Table 1 for in-core depth for this study). At 5 cm, two distinct peaks were identified, DS_137.51 with 60 shards cm^3^ and DS_137.81 with 80 shards cm^3^ (Fig. 2b). Major and minor geochemical analysis of glass shards were undertaken on both of these peaks (see “Methods”). Due to the geochemical results of DS_137.51, the remainder of this paper will focus only on grain-specific glass geochemistry that was obtained from DS_137.51 (n = 51) and further high-resolution sampling at those depths. The geochemical results of shards from DS_137.81 show distinct rhyolitic (n = 13) and a single trachytic chemical composition (see Supplementary Table 1). These compositions do not match any known eruptions from Campi Flegrei but have potential volcanic origins across the Mediterranean (i.e. Pantelleria, Central Anatolia, Hellenic Arc), and so are not discussed any further in this paper.

The glass shards from the 5 cm sample of DS_137.51 are colourless, with a mix of platy with fluted features and cuspate morphologies with a size-range of > 100–25 μm (Fig. 2c). Approximately 85% of these glass shards exhibit altered features, characterised by slightly rounded edges, consistent with previous findings of glass shards in the Dead Sea record^6^. The remaining 15% display unaltered features with sharp edges and distinct morphological characteristics (Fig. 2c).

**Figure 3:**
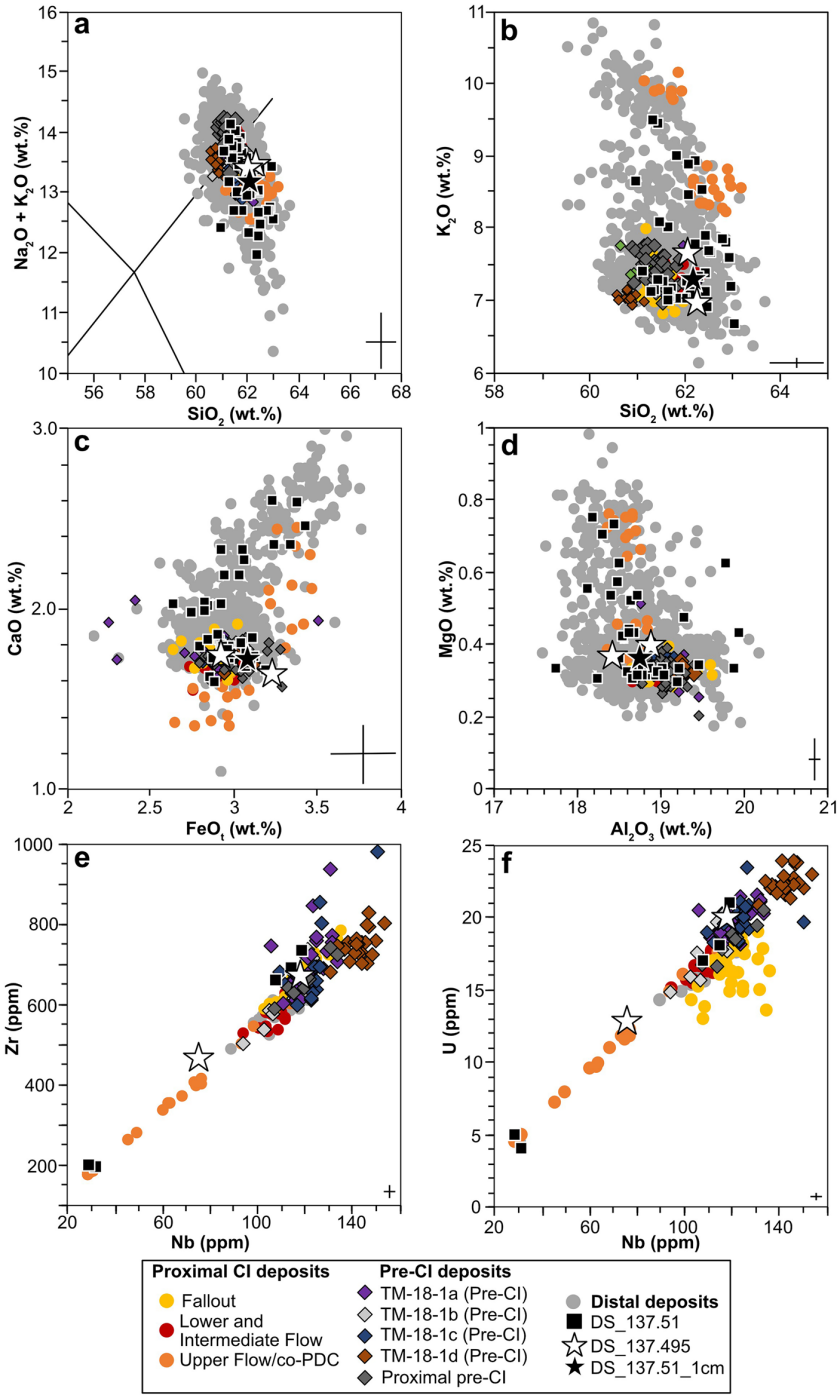
Geochemical analyses of selected glass shards. (**a**) total Alkali Silica (TAS^42^) plot of glass composition from the Dead Sea (DS_137.51, DS_137.495, DS_137.51_1cm) compared to proximal deposits of the fall, lower, intermediate and upper flow deposits^18,19^ as well as distal records^22,24,25,28^. (**b**–**d**) Major element bi-plots showing the glass composition of the shards from the Dead Sea correlate well with proximal and distal CI tephra layers rather than pre-CI deposits. (**e**,**f**) Trace element bi-plots showing selected compositions of the Dead Sea shards and compare well to the composition of the proximal CI^19^, particularly Upper Flow/co-PDC deposits, rather than the pre-CI eruptions^28,43^. Error bars are 2 standard deviations calculated using replica analysis of the MPI-DING StHs6/80-G glass standard^44^ for both major and trace element measurements

The individual geochemical analyses of shards from DS_137.51 are presented in Fig. 3 using selected bi-plots of major and minor elements (data in Supplementary Table 1). Certain glasses from this sample are distinct (n = 46), straddling the phonotrachyic composition with SiO_2_ ranging from 60.99 to 63.06 wt% with high K_2_O (6.64–9.46 wt%), wide ranging Na_2_O (3.33–7.00 wt%) and Na_2_O/K_2_O ratio ranges of 2.57–1.01 wt% (Fig. 3a–d). Trace element analysis was then undertaken to confirm the volcanic source and eruption of these glass shards, particularly as there are several pre-CI eruptions recorded^28^. The analysis of these shards (n = 5) shows wide compositions ranges of 194–732 ppm Zr, 272–486 ppm Rb, 86–283 ppm Ce, 45–155 ppm La, 18–873 ppm Ba, 13–643 ppm Sr, 29–119 ppm Nb, 17–67 ppm Y, 14–64 ppm Th (Fig. 3e,f).

The distinct major and minor chemical compositions of these shards are consistent with the CI eruption from Campi Flegrei, Italy (Fig. 3) and seen in proximal^e.g.^^19^ and distal occurrences^e.g.^^24,25^. The DS_137.51 shards show a large composition scatter, incorporating the different eruption phases of the CI eruption (Plinian and pyroclastic density currents, PDC)^18^. The trace element analysis also provides additional evidence for a correlation to the CI/Y5 proximal deposit, instead of the pre-CI eruptions^19,28^ or other possible volcanic sources in the region (e.g., Aegean Arc^11^).

Based on these geochemical results, the stratigraphic location of the CI was further refined using high-resolution sampling (1–1.5 cm) across the depths of DS_137.51 (see “Methods”). As previously laid out, these depths encompassed a MTD and *aad* sediments^38,39^ (Fig. 2). As the MTD cannot be seen as primary deposited sediment^39^, the *aad* laminations were the focus of this resampling, starting from the depth of 137.475 to 137.51 m blf. Volcanic glasses were found throughout the investigated *aad* depths, with low shard concentrations of 6–8 shards per cm^3^ (Fig. 2). All depths were geochemically investigated for major and minor elements. Two out of the three depths, DS_137.495 and DS_137.510_1cm had two and one shards, respectively, against a background of rhyolitic shards (n = 7, see Supplementary Table 1). These shards have a distinctly different geochemistry and likely originate from other unknown volcanic centres/eruptions from the Mediterranean region (e.g. Anatolia, Hellenic Arch). Since they cannot be related to a certain volcanic eruption, their discussion is beyond the scope of this paper. The important CI shards relevant for our study were a mix of platy with fluted features and cuspate shards at ~ 100 μm in length (Fig. 2c). The shard preservation ranged from altered to unaltered, which is typical of volcanic shards found throughout the Dead Sea record and is due to the high alkalinity of the Dead Sea brine^6^.

These shards (n = 3) underwent geochemical analysis and showed a trachyte composition (SiO_2_: DS_137.495: 62.2 ± 0.1 wt% 1 s.d. DS_137.510_1cm: 62.2 wt%) and lower K_2_O (DS_137.495: 7.3 ± 0.5 wt%; DS_137.510_1cm: 7.2 wt%) (Fig. 3a–d) with Na_2_O/K_2_O ratio ranges of 1.35–1.06 wt% (DS_137.495) and 1.22 wt% (DS_137.510_1cm). Only DS_137.495 underwent trace element analysis due to the sufficient size of the shards (n = 2), providing rare earth element composition identical to the CI with compositional ranges of 467–668 ppm Zr, 376–436 ppm Rb, 175–250 ppm Ce, 103–139 ppm La, 27–35 ppm Ba, 31–39 ppm Sr, 76–118 ppm Nb, 35–54 ppm Y, 38–53 ppm Th (Fig. 3e,f).

The peak in shard concentration at DS_137.495 is defined as the CI isochron, located within a varved (*aad*) interval. The low number of in total three fresh shards identified in the 1 cm samples (DS_137.495 and DS_135.451_1cm) is due to the Dead Sea being at the far edge of the CI ash dispersal^41^. In contrast, the low resolution 5 cm sample DS_137.51, includes a much higher number of CI shards (Fig. 2b) which show clear signs of alteration. This is explained by the fact that the 5 cm sample encompasses the basal part of a major MTD which is reworked sediment from the catchment and littoral zone of the DS. Reworking of CI shards is also witnessed in several other records^24,25^. We can exclude contamination of the two 1 cm samples due to: 1) fresh CI shards are observed in DS_137.495 and DS_135.451_1cm and 2) lack of possible downward shard displacement from the MTD as there are samples in between with no CI shards found.

## Discussion

### Identifying the Campanian Ignimbrite

The geochemical signature of glass shards from the DS record can be confidently correlated to the CI based on major, minor and trace element analysis (Fig. 3). The glasses incorporate the bimodal composition seen at the proximal deposits of the eruption^18,19^ as well as in distal records^24,25^ (Fig. 3). There are close variations in chemical composition that have been related to the different eruption phases from the Plinian eruption and PDC deposits^18^, with different magma chemical compositions tapped during the CI eruption^17–19,45^ (Fig. 3). The fallout, lower and intermediate flow erupted CI tephra  that has a compositional  range across the trachyte-phonolite boundary and Na_2_O/K_2_O ratios below 0.6 wt%, with the Upper flow/co-PDC as trachytic ^18,19^. The CI cryptotephras of DS show more affiliation to the Upper flow/co-PDC deposit due to the higher Na_2_O/K_2_O ratios seen across the 5 cm and 1 cm samples. Many other distally located records also show volcanic glasses with similar chemical compositions^17,24,29^.

By using single glass trace element analysis on the two shards from DS_137.495 and five shards from DS_137.51, further evidence is provided that these shards are from the CI eruption, rather than the multiple smaller pre-CI eruptions seen at a limited number of proximal outcrops^43^ and distal records^25,28^ located in the northern Mediterranean (Fig. 3e and f). Lago Grande di Monticchio has recorded four of these pre-CI tephra layers, TM-18-1a-d, within ~ 600 varve years before the CI which have undergone additional trace element analysis. Recent investigations into pre-CI eruptions at the proximal setting of Acquamorta, Italy have also shown at least seven pre-CI eruption units^43^. The chemical composition of these tephras overlap in major and minor elements with the CI eruption (Fig. 3) with very subtle differences in trace elements^19,28,43^. The CI eruption itself was the largest from Campi Flegrei in 200,000 years with tephra deposits found across the European continent (Fig. 1a), making a distribution to the Dead Sea from this eruption more likely than from any of the smaller pre-CI eruptions.

The low-resolution sample of DS_137.51 shard geochemistry overlaps in composition with the fallout and the different PDC flows including the upper flow unit encompassing the full chemical composition of the CI eruption observed in other distal records^18,19,24,25,29^ (Fig. 3). The number of glasses with high K_2_O composition (~ 50% of the population) in DS_137.51 provides evidence for deposition associated to the co-PDC phase recorded later in the eruption as the caldera collapsed^18^ (Fig. S1). Distal records containing the CI to the northeast and southeast of Campi Flegrei also show co-PDC phase chemical compositions^18^ (Fig. 1).

The discovery of the CI In the DS record significantly extends the known dispersal of this eruption to the south-eastern part of the Mediterranean region, > 2300 km away from Campi Flegrei (Fig. 1). The geochemical association with the co-PDC phase of the eruption shows the higher chances of co-PDC ash reaching the distal Dead Sea due to the larger ash volume produced when compared to the Plinian eruption (~ 154 km^3^ vs ~ 54 km^3^ volume of fallout material^46^), with favourable wind direction and taphonomic processes for the identification and preservation of these shards. The discovery of the CI as a cryptotephra layer is beyond the current modelled isopach maps of the CI ash thickness for the south-eastern ash dispersal direction^46,47^. With DS_137.495 and DS_137.451_1cm having only two and one shards identified as the CI, this may indicate that we might be reaching the most southeastward limits of the plume dispersal for this tephra marker. This discovery highlights  the potential to detect the CI in other palaeoenvironmental and archaeological records in the SE Mediterranean region.

### The chronological implications of the CI

The identification of the CI tephra marker allows the current chronology of the ICDP Dead Sea 5017-1A record to be refined at this depth. The chronology of Goldstein et al.^7^ had used the beginning of the *aad* varves just below the distinct MTD, as an age control point of 40.2 ± 1.6 kya (2σ), using the correlation to the shallow-water deposits of the Lisan Formation nowadays outcropping southwest of the Dead Sea^48,49^. Tierney et al.^28^ used the radiocarbon and U-Th dates close to the CI depth for the recent age-depth model for core 5017-1A. The current refined date for the CI eruption by direct ^40^Ar/^39^Ar dating of proximal deposits is with 39,785 ± 140 cal yr BP^13^ (2σ before CE 1950; 39,925–39,645 cal BP), within the error of the Goldstein et al.^7^ tie-point and Tierney et al.^28^ chronologies. Thus, the integration of the well-constrained, directly dated CI age by Giaccio et al.^13^ enables us to reduce the age uncertainties for the chronology for this part of the Dead Sea record in the future.

### Reconstructing hydroclimatic conditions around the time of the CI eruption

The widespread CI tephra horizon allows correlating the DS with other key palaeoenvironmental records across the Mediterranean (Fig. 1) and investigating the climatic conditions at the time of the eruption across the region. The CI is largely associated with a large-scale climatic downturn with the occurrence of the North Atlantic HE4, leading to a cold and dry phase in the northern and central Mediterranean^31^. This has been recorded in several palaeoenvironmental records of Tenaghi Philippon^30^, Monitcchio^50^, Levos Island^31^ and the Black Sea^21^ (Fig. 1), with the CI falling soon after the beginning of the HE4.

**Figure 4:**
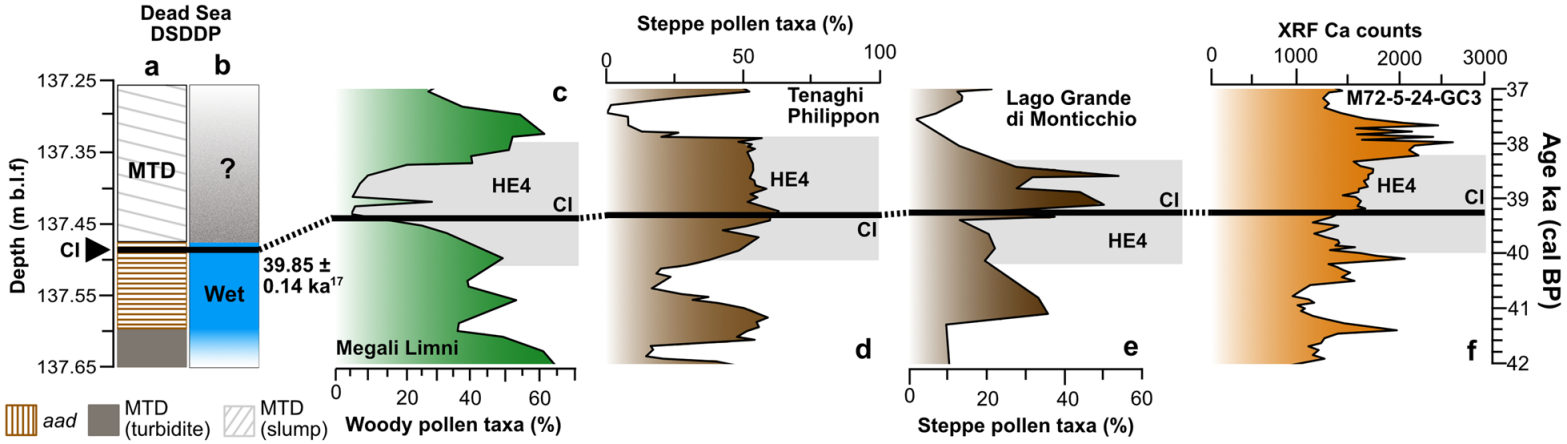
Regional comparison of the stratigraphic position of the Campanian Ignimbrite (solid black line) and the proposed environmental response linked to the Heinrich Event 4 (grey box) in the Mediterranean region. (**a** and **b**) The DSDDP lithological changes with reconstructed climatic conditions with the position of the CI isochrone and the accepted date of the CI; (**c**) Megali Limni pollen record^31^; (**d**) Tenaghi Philippon pollen record^30^; (**e**) Lago di Monticchio pollen record^28,55^; (**f**) The Black Sea Ca XRF-core-scanning record of the core M72/5-25-GC1 Ca-record^21^. Each record is plotted on their own individual timescale with the directly dated age of the CI given as 39.85 ± 0.14ka^13^ for the DSDDP record.

The CI in the Dead Sea record is deposited within *aad* varves (Fig. 2b) which are associated with high lake levels due to higher supply of freshwater, allowing precipitation of primary aragonite^38,40,48,49,51^. The formation of aragonite occurs during the summer while winter flooding provided supply of detrital catchment material, resulting in annual laminations^38,40^. These sediment couplets are seen clearly using XRF with high log Sr/Ca ratios (Fig. 2c). Due to the sampling strategy currently undertaken and also the thinness of the couplets, we cannot confine the CI isochron to either a summer (aragonite) or winter (detrital) layer. Yet, at the time of the CI eruption, the Dead Sea was experiencing wetter conditions. In contrast, in central and eastern Mediterranean sites experienced dry conditions at the time of the CI deposition (Figs. 1a, 4), associated with the HE4 in the region.

Following the deposition of the CI in *aad* varves, a thick MTD unit (> 5 m) is emplaced (Fig. 2a). Though other alternating smaller graded layers (~ 5–7 cm) are also present (defined as turbidites^34^) with *aad* below the targeted sampling interval, this MTD above the CI has been associated to a major slumping event^39^, and tentatively correlated to a missing sediment unit (hiatus) in the Masada onshore outcrop, directly above the so-called “Broken Gypsum Unit” (BGU)^48,49,52^. Previous studies have attributed the BGU to the dry and cold HE4, with a reconstructed lake-level decline of ~ 110 m at the Dead Sea around that time^48,49^, seen across the Dead Sea outcrop sections^48^ but not in the deep-lake record^38^, possibly due to the erosive nature of the MTD. It has been hypothesised that large MTDs (> 50 cm) or slumps were formed due to a sudden change in Lake Lisan’s lake levels as a result of drier climatic conditions^48,49,52^. Further, it has been shown during deglacial times that gypsum units in the deep lake consist of alternating gypsum deposits and MTDs^5^. Thus, the MTD might have been deposited (eroding all gypsum deposits) or directly above. However, the climatic or tectonic origin of this MTD unit in the DSDDP is still debated^7,37,39^. The time-span between the change in wetter conditions (*aad* varves) to the event conditions (MTD/slump) experienced at the Dead Sea cannot be fully quantified due to the erosive contact between the layers (Fig. 2), even with the CI isochron. However, a minimum of around 16 varve years have been counted from the isochron to the overlying MTD unit. Nonetheless, it is clear that the CI has been identified when the Dead Sea experienced wetter conditions.

### A time-transgressive response of Mediterranean climates to HE4

During the CI eruption, the central and eastern Mediterranean were experiencing cold and dry conditions, coinciding with H4 as reconstructed from numerous palaeoenvironmental records (Fig. 4)^49,53,54^.

During HE4, the influx of freshwater into the North Atlantic inhibited the North Atlantic Deep-water formation, resulting in cold sea surface temperatures in the Mediterranean Sea^52^. Coupled with colder air masses that are brought southward by the southward displacement of synoptic atmospheric systems, such as the polar front^54^, evaporation was reduced leading to increased aridification across the eastern Mediterranean region as storm and rainfall frequency decreased^48,49,52^. The correlation of Heinrich Events in the North Atlantic has been assumed to be synchronous with aridification in the Eastern Mediterranean^49,53,54^.

In contrast, the Dead Sea experienced wetter environmental conditions at the time of the CI eruption. This strongly suggests local delays in the DS region to the overall aridification around that time. The southward shift of atmospheric circulation may have been gradual with the central and eastern Mediterranean sites experiencing aridification before the most southern site of the Dead Sea (Fig. 4). Similar climatic transitions have been witnessed, using tephra as a time marker, in northern Europe during the Younger Dryas (~ 12 kya)^8^. No accurate offset between the wetter conditions at the Dead Sea and the HE4 can be quantified due to the erosion of the *aad* varves in the DSDDP. Yet, a minimum of ~ 16 years using the *aad* varves is estimated. Nonetheless, this proposed delayed local environmental response can be seen as a time-transgressive transition to the HE4 as oceanic-atmospheric systems took time to reorganise, rather than a synchronous response across the entire Mediterranean region.

The high sediment accumulation in the 5017-1A core has provided a valuable detailed archive. The identification of important and useful tephra can improve the existing age model for the 5017-1A core and significantly reduce age uncertainties, providing valuable temporal and regional insights into environmental response to past abrupt climate change. The identification of the important and well-dated CI tephra in an interval of *aad* varves has proven that regional differences of wet/dry conditions occur in the Eastern Mediterranean during HE4.

## Summary and concluding remarks

Our new discovery of a CI cryptotephra layer in Dead Sea record in the eastern Mediterranean region, confirmed through major, minor and trace element analysis, extends this important tephrostratigraphic marker further into the south-eastern part of the Mediterranean region. This is the first time that this valuable tephra layer, preserved as a cryptotephra layer here, has been found in the Levant, over 2300 km away from its volcanic source. This discovery provides an invaluable absolute chronological marker for the Dead Sea record, which has unambiguous age control points with large uncertainties. The identification of the CI provides a direct tie point to precisely synchronise the Dead Sea to other palaeoclimatic records over the Mediterranean region. The instance of the CI within the Dead Sea record at the time of *aad* varve formation indicates wet conditions and high lake levels, soon followed by a destructive MTD unit. This is different to the climate recorded in central and eastern Mediterranean palaeoenvironmental records at the time of the CI eruption, where the climate conditions were already dry in response to the H4 cold phase in the N Atlantic. The use of the continental scale CI tephra marker has questioned the assumption that the Eastern Mediterranean is responding synchronously to abrupt climatic changes and can be further examined in future investigations with other well-dated, high-resolution palaeoenvironmental archives in the southern extent of the Eastern Mediterranean. This work shows the temporal and spatial complexity of regional responses to global climatic changes, and emphasises the value of tephra layers as isochrons. 

## Methods

### Sample selection

Samples from the core 5017-1-A-59-1 were investigated for preservation of cryptotephra due to being identified within the age range of the CI eruption, as defined by Giaccio et al.^13.^ The relevant depth range was established through a combination of:  (1) previous sedimentological investigations from Torfstein et al.^48,49^; (2) the re-calibrated age model results from Tierney et al.^28^ using IntCal20^56^ and; (3) the tie point ages given by Goldstein et al.^7^. As a result, the Core 59 Section 1 of this core was identified as the best part of the DS record to investigate for the CI. Due to the number of conflicting depths used in numerous publications from the ICDP Dead Sea DSDDP record, the depth used in this paper is given as drilling depths (m b.l.f). Additional information is given in Supplementary Table 1 on the depth in the core section.

### Tephra sample preparation

Continuous 5 cm resolution sampling was undertaken across the sediment depths that were identified to be within the age range of the CI eruption as described above on the working half of the 5017-1A core. Samples that were entirely in MTD units (> 5 cm) were excluded due to being clearly reworked. The glass shards were extracted from the wet sediment using the following methods. Due to the high salt content of the Dead Sea sediments, additional steps were taken following Neugebauer et al.^6^. The sediment was first washed with deionised water in a shaking bath to remove as much as salt as possible. The samples were then treated with 10% HCl to remove carbonates and then 15% H_2_O_2_ to remove organic matter, followed by wet sieving at 20–100 μm grain-size fraction. The remaining sediment fraction underwent the density separation procedure following Blockley et al.^57^ using sodium polytungstate (SPT). The cryptotephra was concentrated at 2–2.55 g cm^−3^ density fraction. Additional sieving was undertaken using a 20 μm mesh to remove any excess clay residue. Due to the high amount of remaining sediment at the end of the procedure, *Lycopodium* spores of known concentration (batch no. 050220211, Laboratory of Quaternary Geology, University of Lund, Sweden) were added to each sample. A total of at least 5–10% of each sample were then mounted on glass shards using UV resin. Volcanic glass shards were then identified using a high-powered, polarising optical microscope. Where a peak in glass shard concentration was identified, the remaining ~ 90% of that sample was picked for ~ 30 individual glass shards using a micromanipulator^58^, dried, then sealed in epoxy resin. This sample was then sectioned to expose the glass shards on the surface and polished to undergo geochemical analysis.

Due to the results of the geochemical analysis of DS_137.51 (as explained above), the *aad* laminated depths of this sample were continuously resampled at high-resolution of 1–1.5 cm (to incorporate all of the laminated depths within DS_137.51 only) on the working half of the 5017-1A core. These samples underwent the same modified extraction technique detailed above from Blockley et al.^57^ and Neugebauer et al.^6^. Due to the low amount of remaining sample after this procedure, no *Lycopodium* was added. Each sample was then completely scanned for volcanic glass shards in water using a digital microscope, the Keyence Digital Microscope VHX-970F, counted and each of the shards identified were directly picked from water using a micromanipulator straight away. The use of a digital microscope and application of counting and picking in water at the same time, allowed for a fast and complete recovery of all the glass shards identified in each finite sample to be used for geochemical analysis.

### Geochemical analysis

#### Major and minor element analysis (EPMA)

Major and minor element compositions of the glass shards identified and picked from each sample were measured using an EPMA. For the DS_137.51 glass shards, the measurements were conducted on a wavelength-dispersive JEOL JXA-8200 microprobe at the University of Potsdam, Potsdam. The instrument conditions at the University of Potsdam were 15 kV voltage and a 10 nA beam current for the beam sizes 5-10 μm. The count times were 20 s for Mg, Ti, F, P, Mn and Cl, 10 s for Fe, Si, Al and Ca and 6 s for Na and K. The secondary glass standards run along the glass shards samples were MPI-DING glasses of StHs6/80-6 and GOR128-G^44^, Lipari obsidian^59^ and the Smithsonian Institute VG-568^60^.

For the high-resolution samples, a JEOL JXA-8500F electron microprobe was used at the GFZ, Potsdam. The instrument settings were 15 kV voltage, 5nA beam current with a beam size of 5–10 μm due to the mainly vesicular nature of the glass shards limiting the exposed surface area for analysis. The count times of 20 s for the elements Fe, Cl, Mn, Ti, Mg and P and 10 s for F, Si, Al, K, Ca and Na. Secondary glass standards from the MPI-DING glasses, ATHO-G, StHs6/80-6 and GOR128-G^44^ along with Lipari obsidian^59^ were used to monitor precision and accuracy of the measurements.

Verification between the University of Potsdam’s EPMA and GFZ’s EPMA were done through the same type of standards used on both machines (StHs6/80-6, GOR128-G and Lipari) and a previously measured homogenous internal (‘unknown type’) sample on GFZ EPMA. Little variation is seen been the two different EPMA’s and so both results and comparison of data is deemed conclusive.

Analysis with analytical totals of < 92% were discarded. The data was normalised to 100% after the removal of volatiles (water, F and Cl) and is presented as such in this paper. Error bars presented within the graphs are calculated as 2 × Standard Deviations of the StHs6/80-G MPI-DING. All non-normalised major and minor element data are presented in full in the supplementary file 1.

#### Trace element composition analysis (LA-ICP-MS)

Trace element analysis was performed on selected glass shards from the low-resolution sample of DS_137.51 and the high-resolution sample of DS_137.51_1cm. The shards were selected on their size being big enough for a 20 μm beam size to provide a signal long enough to produce reliable results. This resulted in a limited number of shards (n = 2) meeting these criteria in DS_137.51_1cm.

The general information regarding the LA-ICP-MS trace element analyses of tephra samples can be found in Tomlinson et al.^61^. The analysis in this study were conducted at GEOMAR Kiel, Kiel, on the Nu instruments ATTOM HR-ICP-MS coupled to a NWR UP193fx laser ablation system following the approach of Fietzke and Frische^62^. The ICP has been operated under hot plasma conditions (normalized Argon index NAI ~ 30 and ThO/Th ~ 0.04%) using 1100 W rf power, 17.5 l/min cool gas, 0.6 l/min auxiliary gas, 0.6 l/min Ar sample gas mixed with 0.7 l/min He cell gas from the laser unit. The laser was operated at 5 Hz repetition rate using a fluence of 3.1 J/cm^2^ and a spot size of 20 µm. Prior to each ablation interval 40 s of gas blank data had been collected while the laser was warming up. The laser actively ablated for 40 s followed by 15 s of wash out. All samples and standards were pre-ablated by 5 shots using a spot diameter of 35 µm. The analytical run included several analyses of unknown glasses and bracketed by MPI-DING (ATHO-G, StHs6/80-6 and GOR128-G^44^), Lipari obsidian^59^ and NIST612 and NIST610 (GeoREM 11/2006) for calibration. 29Si has been used for internal normalisation. ^27^Al, determined by previous EPMA analysis has been used for the conversion to TE/Si data into TE concentration values. Microsoft Excel has been utilised for the entire data reduction.

#### Microfacies analyses and micro-XRF measurements

A detailed examination of the microfacies was performed on a sediment block of 10 cm-long, 2 cm-wide and 1 cm-thick that was cut out of the fresh sediment on the archive half of the 5017-1A core due to the lack of continuous preservation of sediment in the working half. As a result, the sediments are representative of the working half but mirrored. Preparation of thin-sections from soft and wet sediment blocks followed a standard procedure minimizing process-induced disturbances of sediment micro-structures and included shock-freezing with liquid nitrogen, freeze-drying for 48 h, and epoxy resin impregnation under vacuum^63^.

Detailed microfacies analysis was performed on large-scale petrographic thin sections. Microscopic analysis included the investigation of sediment using a petrographic microscope with non-polarized and (cross)-polarized lights, at 5×–40× magnifications (Carl Zeiss Axioplan).

The epoxy embedded sediment block (5017-1A-59-1A) was used for µ-XRF element mapping. Measurements are conducted every 50 µm at 50 kV, 600 µA and 50 ms using a Bruker M4 Tornado, which is equipped with a Rh X-ray source in combination with poly-capillary X-ray optics generating an irradiation spot of 20 µm. Mapping results represent sediment layer of detrital (Si), calcium carbonate (ca) and aragonite (Ca + Sr) and gypsum (Ca + S). However, elements that occur predominantly in pore fluids (e.g., Cl and S) are not well preserved in epoxy-embedded samples.

### Supplementary Information

Supplementary Figure S1.

Supplementary Tables.

## Data Availability

All data generated during this study are included in this published article and is available in Supplementary Information Tables S1–S4.
