# Supplementary material for: Identification of the Campanian Ignimbrite in the Dead Sea and consequent time-transgressive hydroclimatic shifts in the Eastern Mediterranean
Source: Sci Rep. 2024 May 27;14:12114. doi: 10.1038/s41598-024-59639-7 (PMC11130199; doi:10.1038/s41598-024-59639-7)
Supplement: Supplementary file 1 — Supplementary Figure S1. [file 41598_2024_59639_MOESM1_ESM.docx]

**Identification of the Campanian Ignimbrite in the Dead Sea and consequent time-transgressive hydroclimatic shifts in the Eastern Mediterranean**

Rebecca J. Kearney^1^, , Markus J. Schwab^1^, Daniel Redant^1^, Ina Neugebauer^1^, Oona Appelt^2^, Cecile Blanchet^3^, Jan Fietzke^4^, Christina Günter^5^, Daniela J.M. Müller^1,6^, Rik Tjallingii^1^, Achim Brauer^1,5^

**Supplementary information**

**Figure S1**


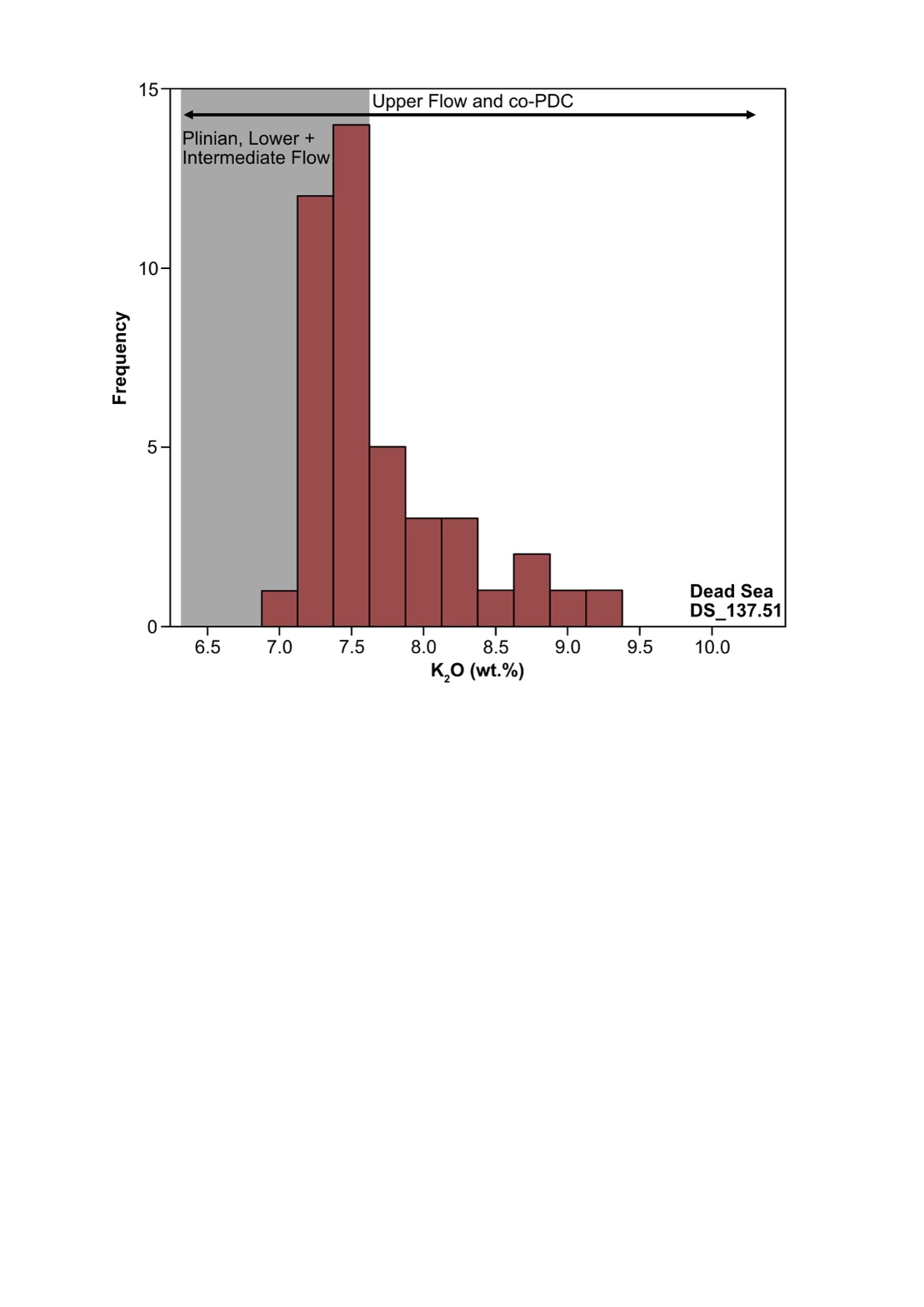


**Fig. S1.** Histogram of the K_2_O glass composition of the DS_137.51 low-resolution Campanian Ignimbrite shards. Following Smith et al.^[1]^, the grey shaded box shows the more evolved compositions of the early Plinian and Lower Flow deposit. The wide distribution of the K_2_O values from the Dead Sea shows the CI shards incorporate the same chemical composition from the Upper flow and co-PDC proximal deposits rather than just the composition associated with the earlier eruption’s phases of the Plinian and Lower Flow deposit. This distinct chemical signature provides further evidence that the Dead Sea cryptotephra is from the CI eruption rather than pre-CI which have a narrower chemical range.

**References**

1. Smith, V. C., Isaia, R., Engwell, S. L. & Albert, P. G. Tephra dispersal during the Campanian Ignimbrite (Italy) eruption: implications for ultra-distal ash transport during the large caldera-forming eruption. *Bull Volcanol* **78**, (2016).
